# Genetic Diversity and Population Structure of the Critically Endangered Yangtze Finless Porpoise (*Neophocaena asiaeorientalis asiaeorientalis*) as Revealed by Mitochondrial and Microsatellite DNA

**DOI:** 10.3390/ijms150711307

**Published:** 2014-06-25

**Authors:** Minmin Chen, Jinsong Zheng, Min Wu, Rui Ruan, Qingzhong Zhao, Ding Wang

**Affiliations:** 1 The Key Laboratory of Aquatic Biodiversity and Conservation of Chinese Academy of Sciences, Institute of Hydrobiology of Chinese Academy of Sciences, Wuhan 430072, China; E-Mails: chenminminok@163.com (M.C.); wumin_926@163.com (M.W.); ruan.rui@163.com (R.R.); zhaoqz0517@163.com (Q.Z.); 2 University of Chinese Academy of Sciences, Beijing 100049, China

**Keywords:** Yangtze finless porpoise, genetic diversity, genetic structure, population fragmentation, genetic conservation

## Abstract

Ecological surveys have indicated that the population of the critically endangered Yangtze finless porpoise (YFP, *Neophocaena asiaeorientalis asiaeorientalis*) is becoming increasingly small and fragmented, and will be at high risk of extinction in the near future. Genetic conservation of this population will be an important component of the long-term conservation effort. We used a 597 base pair mitochondrial DNA (mtDNA) control region and 11 microsatellite loci to analyze the genetic diversity and population structure of the YFP. The analysis of both mtDNA and microsatellite loci suggested that the genetic diversity of the YFP will possibly decrease in the future if the population keeps declining at a rapid rate, even though these two types of markers revealed different levels of genetic diversity. In addition, mtDNA revealed strong genetic differentiation between one local population, Xingchang–Shishou (XCSS), and the other five downstream local populations; furthermore, microsatellite DNA unveiled fine but significant genetic differentiation between three of the local populations (not only XCSS but also Poyang Lake (PY) and Tongling (TL)) and the other local populations. With an increasing number of distribution gaps appearing in the Yangtze main steam, the genetic differentiation of local populations will likely intensify in the future. The YFP is becoming a genetically fragmented population. Therefore, we recommend attention should be paid to the genetic conservation of the YFP.

## 1. Introduction

**Figure 1:**
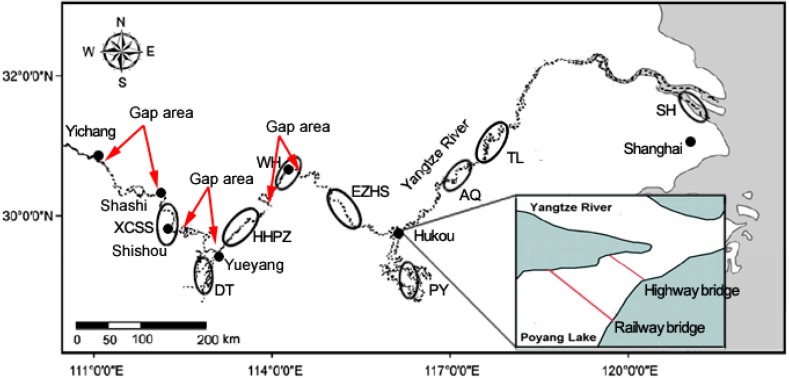
The black circles represent sampling localities of the Yangtze finless porpoise in this study. Arrows indicate the gap areas discovered by the Yangtze Freshwater Dolphin Expedition. SH = Shanghai, TL = Tongling, AQ = Anqing, PY = Poyang Lake, EZHS = Ezhou–Huangshi, WH = Wuhan, HHPZ = Honghu–Paizhou, DT = Dongting Lake, XCSS = Xingchang–Shishou.

The Yangtze finless porpoise (YFP, *Neophocaena asiaeorientalis asiaeorientalis*) which was reclassified as a subspecies of the narrow-ridged finless porpoise (*N. asiaeorientalis*) in 2009 [1], inhabits only the middle and lower reaches of the Yangtze River (from Yichang to Shanghai) and the adjoining Poyang and Dongting Lakes [2] (Figure 1). As a mammalian species at the top of the food chain, its survival depends heavily on habitat stability and food resource availability [3]. However, over-exploitation of the Yangtze River by many types of human activities has led to pollution, habitat loss, habitat fragmentation and food shortage, which seriously threaten the survival of the YFP [3,4]. The YFP population has declined remarkably over the last two decades, especially in the Yangtze main stream, decreasing from more than 2500 individuals in 1991 [5] to fewer than 1225 in 2006 [6]. According to the 2006 Yangtze Freshwater Dolphin Expedition (YFDE2006), the total population size, including those in the Poyang and Dongting Lakes, was approximately 1800 [6], whereas the 2012 Yangtze Freshwater Dolphin Expedition (YFDE2012) revealed that at the end of 2012, there were only approximately 1040 individuals remaining, including 500 in the Yangtze main stream, 450 in the Poyang Lake and 90 in the Dongting Lake [7]. Owing to the small population size, sharply declining population, and high probability of extinction, the YFP was recently reclassified as a Critically Endangered population on the IUCN (International Union for Conservation of Nature and Natural Resources) Red List [8]. In fact, the YFP population is suffering not only from a sharp decline in number but also from population fragmentation. A distribution gap of approximately 150 km between Shishou and Yueyang was first revealed by the YFDE2006, during which no porpoises were found in this region [6] (Figure 1). Two additional gaps were found by YFDE2012 (in the section between Yichang and Shashi, and a section of approximately 150 km around Wuhan) [7] (Figure 1). Population fragmentation may also have occurred between the Yangtze main stream and the Poyang and Dongting Lakes owing to the high-density human activities in these estuary areas over the past few years [9,10]. For example, in the Dongting Lake, high-density of sand-transport vessels nearly occupy all the estuary areas. In the Poyang Lake, besides the effect of sand-transport vessels, the construction of two large bridges across the estuary area (a highway bridge in 2000 and a railway bridge in 2008) (Figure 1) also seriously affect the river-lake migration of the YFP. Studies showed that the historical large-scale river-lake migration of the YFP has not occurred since the construction of the two bridges [9,11]. Overall, the ecological surveys indicated that the YFP population was becoming increasing small and fragmented.

A good knowledge of the genetic diversity and structure in endangered species is a necessary prerequisite for conservation, especially for those that suffer from fast decline and fragmentation, as it reflects the status and survival potential of populations [12,13,14,15]. A previous study found that the mitochondrial genetic diversity of the YFP is low (*h* = 0.65, π = 0.0011), and significant genetic differentiation had developed between the XCSS population and the other five downstream local populations, based on the analysis of the complete mtDNA control region from 39 porpoises [16]. Low mtDNA genetic diversity of the YFP population (*h* = 0.52, π = 0.0017) was also detected by Yang *et al*. [17], based on the analysis of a 345 base pair mtDNA control region with an increased sample size of 105 porpoises. However, these studies may not accurately reflect the genetic diversity and population structure of the YFP population because mtDNA represents only a single locus and reflects only the history of the female lineages. In addition, previous studies all focused on the YFP population in the Yangtze main stream, and very little information was obtained regarding the genetic diversity and genetic differentiation of porpoises living in the Poyang and Dongting Lakes, which support nearly half of the total population. Loss of genetic variation and genetic differentiation may have already occurred as the population has continued to decline and become increasingly fragmented. Therefore, in this study, we combined mtDNA control region analysis with an analysis of bi-parentally inherited microsatellite DNA and extensive sampling at localities including not only the distribution range in the Yangtze main stream but also the adjacent Poyang and Dongting Lakes. This approach allowed us to (i) estimate the current genetic diversity of the YFP and its distribution across the entire range; (ii) detect possible genetic differentiation between the different local populations; and (iii) explore the evolution of genetic diversity in this population under different demographic scenarios using computer simulations. We then provide recommendations for genetic conservation and management of this critically endangered population based on our results.

## 2. Results

### 2.1. Genetic Diversity of the Yangtze Finless Porpoise

Five hundred and ninety-seven base pair of mtDNA control region sequences were obtained from 168 individuals, while the sequences of the other 18 porpoises failed to be amplified due to poor DNA quality. In total, eight variable sites were detected and eight haplotypes were defined as NAACR-Hap1–NAACR-Hap8 with GenBank accession numbers KC135874–KC135881 (Table 1). Among the eight mitochondrial haplotypes, NAACR-Hap1 and NAACR-Hap2 are the main haplotypes that are widely distributed across the entire population and were shared by 55 and 98 individuals in our study, respectively, whereas the other six are rare haplotypes that were detected in very few individuals. For example, NAACR-Hap5 was only found in five individuals within three of the local populations (Wuhan (WH), Honghu–Paizhou (HHPZ), and Dongting Lake (DT)). However, the other five haplotypes occurred sporadically and were limited to a specific local population (Table 1, Figure 3). The haplotype and nucleotide diversities of the entire population were 0.55 ± 0.03 and 0.0011 ± 0.0009, respectively, and are shown in Table 2. The two limnic populations (Poyang Lake (PY) and DT) contained two main haplotypes and two rare haplotypes, representing 50% of the mtDNA haplotype diversity.

**Table 1:** Variable sites detected in the 597 base pair mtDNA control region of the Yangtze finless porpoise, including haplotypes definitions and their distribution among local populations.

Haplotype	Variable Site								Distribution of Haplotypes									
	0	1	1	2	3	3	3	5										
	3	0	8	1	0	0	6	0	SH	TL	AQ	PY	EZHS	WH	HHPZ	DT	XCSS	Total
	0	5	5	8	0	1	7	8										
NAACR-Hap1	C	C	C	A	A	T	T	G	0	3	0	30	4	0	3	3	12	55
NAACR-Hap2	T	.	.	.	.	.	.	.	2	11	2	56	7	2	9	9	0	98
NAACR-Hap3	T	.	.	.	.	.	.	-	0	2	0	0	0	0	0	0	0	2
NAACR-Hap4	.	A	.	G	.	.	.	.	0	0	0	0	1	0	0	0	0	1
NAACR-Hap5	.	.	.	.	.	C	.	.	0	0	0	0	0	1	2	2	0	5
NAACR-Hap6	T	.	T	.	.	.	.	.	0	0	0	0	1	0	0	0	0	1
NAACR-Hap7	T	.	.	.	G	.	.	.	1	0	0	0	0	0	0	0	0	1
NAACR-Hap8	T	.	.	.	.	.	C	.	0	0	0	5	0	0	0	0	0	5

NAACR-Hap1–NAACR-Hap8 were eight haplotypes, “**.**” indicates that the base in this locus is the same as in NAACR-Hap1.

**Table 2:** Genetic diversity of the local Yangtze finless porpoise populations revealed by mtDNA and microsatellite data.

Locality	Microsatellite DNA						Mitochondrial DNA		
	*n*	*N*	*AR*	*H*o	*H*e	*F*is	*n*	*h*	π
Total	186	8.18	5.104	0.663	0.665	−0.006 ^NS^	168	0.55 ± 0.03	0.0011 ± 0.0009
TL	17	5.36	4.617	0.699	0.642	−0.019 ^NS^	16	0.51 ± 0.13	0.0009 ± 0.00076
PY	91	6.82	4.561	0.626	0.644	0.029 ^NS^	91	0.52 ± 0.04	0.0009 ± 0.00048
EZHS	15	5.27	5.230	0.649	0.631	−0.054 ^NS^	13	0.65 ± 0.11	0.0016 ± 0.00129
HHPZ	14	5.45	5.271	0.663	0.702	−0.061 ^NS^	14	0.56 ± 0.13	0.0013 ± 0.00079
DT	20	6.09	5.274	0.710	0.702	−0.066 ^NS^	14	0.56 ± 0.13	0.0013 ± 0.00079
XCSS	16	5.27	4.582	0.696	0.649	−0.083 ^NS^	12	0	0

Number of alleles detected per locus (*N*), allelic richness (*AR*), observed (*H*o) and expected (*H*e) heterozygosity, inbreeding coefficient (*F*is), number of individuals scored (*n*), haplotype diversity (*h*) and nucleotide diversity (π). NS = not significant.

**Figure 2:**
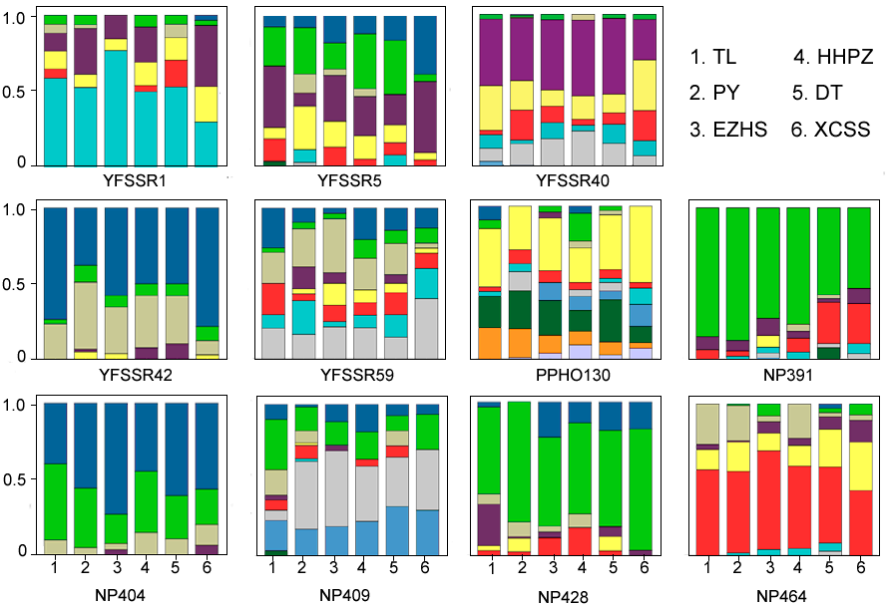
Distribution of genetic diversity at each microsatellite locus across six local populations. The different colors in each box represent different alleles.

A total of 92 alleles were detected at the 11 microsatellite loci from the 186 animals. No evidence was found for null alleles, stuttering, or allele dropout at any of the loci using the program MICRO-CHECKER, with a confidence level of 95%. Allelic number varied from six to twelve per locus with a mean of 8.18, and the overall *AR* was 5.104. The overall *H*o and *H*e were 0.663 and 0.665, respectively. For each local population, the *AR* ranged from 4.561 (PY) to 5.274 (DT), *H*o ranged from 0.626 (PY) to 0.710 (DT) and *H*e ranged from 0.631 (Ezhou–Huangshi (EZHS)) to 0.702 (HHPZ and DT) (Table 2). Genetic diversity of each locus in each local population was showed in Table S2. The distribution of alleles across the local populations is shown in Figure 2. The two limnic populations (PY and DT) contained 85% of the total allelic diversity (78 alleles across 11 loci). No deviation from Hardy-Weinberg equilibrium was detected for any of the loci in any of the local populations except for the YFSSR5 locus in the PY population. No heterozygote excess was found under SMM and TPM (*p* > 0.05) for any of the local populations, and the distribution of allele frequency was L-shaped, as expected in populations under mutation-drift equilibrium. No inbreeding was observed in any of the local populations (*p* > 0.05, Table 2).

### 2.2. Genetic Differentiation of the Yangtze Finless Porpoise

**Figure 3:**
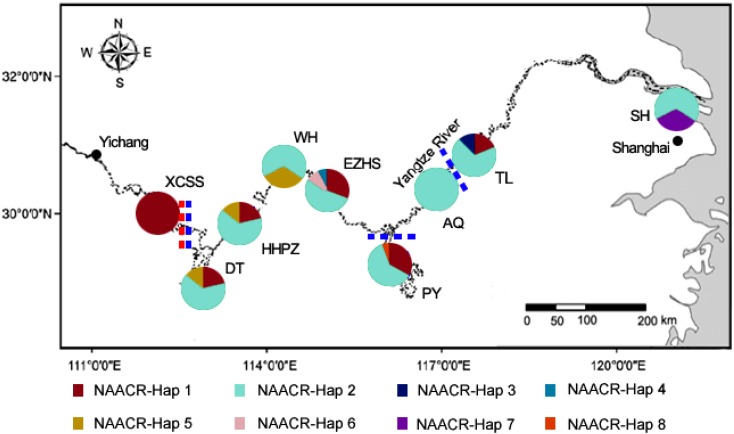
Pie charts show the spatial distribution of the different mtDNA haplotypes. Blue and red dotted line marks the genetic structure, as revealed by the *F*st values based on microsatellites and mtDNA, respectively.

The significant *F*st values obtained by mtDNA ranged from 0.412 to 0.669 (Table 3) with an average of 0.507, and resulting in significant genetic differentiation between the XCSS local population and the five other downstream local populations (TL, PY, EZHS, HHPZ, and DT) (Figure 3). Moreover, the *F*st analysis based on microsatellite data revealed fine but significant genetic differentiation between two additional local populations (not only XCSS but also PY and TL) and the others with *F*st values ranging from 0.025 to 0.058 (Table 3). Thus, the entire YFP population could be divided into four parts (*i.e.*, XCSS, DT–HHPZ–EZHS, PY, and TL) (Figure 3).

**Table 3:** Pairwise *F*st estimates based on the mtDNA and microsatellite data.

	TL	PY	EZHS	HHPZ	DT	XCSS
**TL**		0.032	0.027	0.032	0.032	0.669 ***
**PY**	0.038 ***		−0.004	0.001	0.001	0.466 ***
**EZHS**	0.030 *	0.031 ***		−0.030	−0.030	0.412 ***
**HHPZ**	0.018	0.042 ***	−0.003		−0.077	0.495 ***
**DT**	0.027 *	0.042 ***	0.004	−0.005		0.495 ***
**XCSS**	0.046 ***	0.058 ***	0.032 *	0.031 *	0.025 **	

Above diagonal, pairwise *F*st values based on mtDNA data; below diagonal, pairwise *F*st values based on microsatellite data; the significance indicated is after the Bonferroni correction. * *p* < 0.05, ** *p* < 0.01, *** *p* < 0.0001.

STURCTURE analysis revealed an average maximum log likelihood of posterior probability at *K* = 3 in ten independent simulations (Figure S1a). However, the Var[Ln *P*(*D*)] values were very large at *K* = 3, which indicated that *K* = 3 was not the optimal clustering (Table S1, Figure S2). While at *K* = 1, although the Ln *P*(*D*) values were slightly smaller, they were very stable (Table S1) indicating that all individuals were likely to be assigned to one cluster. Δ*K* failed to find the best *K* which may be due to the lack of subpopulation division (Figure S1b) [18].

**Figure 4:**
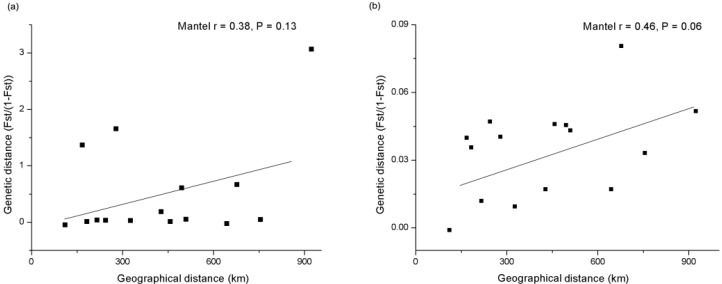
Isolation by distance analysis based on mtDNA (**a**) and microsatellite data (**b**).

Isolation by distance analysis detected no significant correlation between geographical distances and genetic distances for mtDNA data (*r* = 0.38, *p* = 0.13; Figure 4a). While marginal significant correlation was detected for microsatellite data (*r* = 0.46, *p* = 0.06; Figure 4b) suggesting landscape features may have some impact on the genetic differentiation based on microsatellite data, but not strictly significant.

### 2.3. Simulations of the Evolution of Genetic Diversity in the Yangtze Finless Porpoise

**Figure 5:**
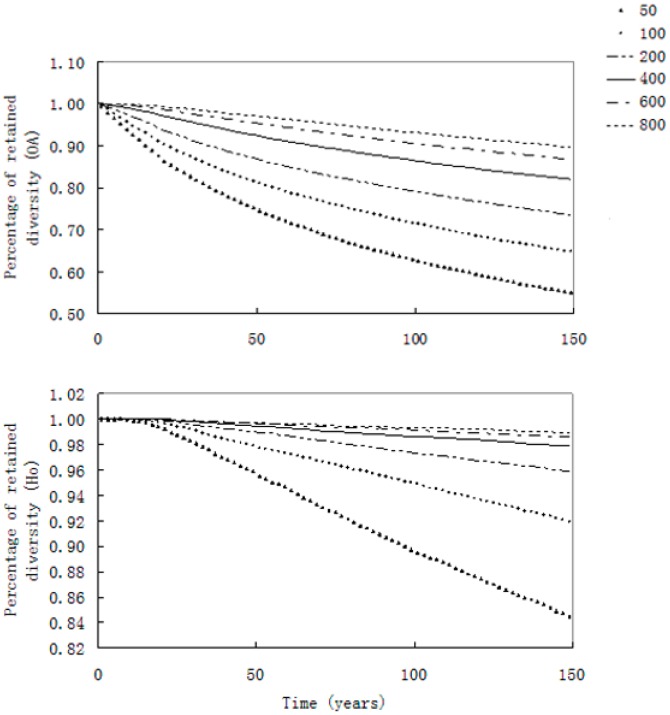
Evolution of the microsatellite genetic diversity over a 150-year period according to BOTTLESIM simulations, assuming different *N*e values held constant over time with the same initial genetic diversity. *OA*, observed number of alleles; *H*o, observed heterozygosity. According to the percentage of the retained number of alleles after 100 years, the minimal effective population size of the Yangtze finless porpoise is approximately 600.

The simulated levels of genetic diversity (the observed number of alleles (*OA*) and observed heterozygosity (*H*o)) that would be retained after a 150-year period, with a stable population size characterized by an initial genetic diversity equal to the level we observed in the empirical data, showed that the allelic diversity declined considerably faster than *H*o (Figure 5). Therefore, the effective population size would need to remain approximately 600 to achieve the general conservation goal of retaining 90% of the genetic diversity over a 100-year period, in which case 90.59% of the genetic diversity would be preserved. 

## 3. Discussion

The YFP has been listed as Critically Endangered by the IUCN Red Book because of its small population size, accelerating population decline and high risk of extinction [8]. Furthermore, according to the YFDE2012, an increasing number of distribution gaps appeared in the Yangtze main stream, suggesting that the YFP is becoming a fragmented small population. Loss of genetic diversity and population genetic structure may occur easily in a fragmented, small population, which may increase the risk of extinction associated with the demographic change [12]. In the present study, a detailed assessment of the genetic diversity and genetic structure of the YFP was performed to investigate its genetic status. Our study suggested that the genetic diversity of the YFP will possibly decrease in the future along with the rapid population decline, based on the results of both mtDNA and microsatellite loci, even though these two types of markers revealed different levels of diversity. MtDNA revealed that the genetic diversity of the YFP was low (*h* = 0.55, π = 0.0011) compared with the finless porpoise population in the Yellow Sea (*h* = 0.8391, π = 0.0036, [19]) and some other cetacean species (e.g., the harbor porpoise, *Phocoena phocoena*, *h* = 0.93, π = 0.011, [20]; the short-beaked common dolphin, *Delphinus delphis*, *h* = 0.949–0.968, π = 0.018, [21]; and the Dall’s porpoise, *Phocoenoides dalli*, *h* = 0.968, π = 0.0106, [22]), which is consistent with the results of previous studies [16,17]. Furthermore, the distribution pattern of the mtDNA haplotypes indicated that aside from the two main haplotypes (NAACR-Hap1 and NAACR-Hap2), the six rare haplotypes were shared by only 15 individuals, among which the NAACR-Hap4, NAACR-Hap6, and NAACR-Hap7 haplotypes were each only found in a single individual (Table 1). With the rapid decline of the YFP population, especially in the Yangtze main stream, the rare mtDNA haplotypes will be easily lost. Therefore, the mtDNA diversity of the YFP is likely to decline in the future. In contrast, the microsatellite DNA analysis (*H*e = 0.665) indicated that the current nuclear genetic diversity of the YFP was moderate compared with the marine finless porpoise populations (*H*e = 0.780–0.795, [23]) and some cetacean species (e.g., the short-beaked common dolphins, *Delphinus delphis*, *H*e = 0.687–0.705, [21]; the bottlenose dolphin, *Tursiops truncatus*, *H*e = 0.624–0.720, [24]; and the Eastern Tropical Pacific spotted dolphin, *Stenella attenuata*, *H*e = 0.694–0.784, [25]). The lack of significant heterozygote excess and the L-shaped allele frequency distribution pattern in all local populations suggested that the recent contraction of the YFP population did not lead to a rapid loss of alleles. However, the results of the simulations based on the microsatellite analysis suggested that a minimum effective population size of approximately 600 is necessary to retain 90% of the current genetic diversity of the YFP over the next 100 years. However, the YFDE2012 revealed that the total size of the YFP was only approximately 1040 with an accelerating decline rate, and the estimated effective population size of the YFP was only approximately 360 at the present time [26]. Thus, the nuclear genetic diversity of this population will likely be lost in the future, because the simulations indicated that a smaller effective population size will experience a more rapid loss of genetic diversity (Figure 5).

Furthermore, significant population genetic differentiation has been observed in the YFP, which will further exacerbate the loss of genetic diversity. For the Yangtze main stream, the results from both the mtDNA and microsatellite analysis supported a significant genetic differentiation between the XCSS and other local populations based on the *F*st values (Table 3). This result is consistent with Zheng *et al*. [16], who found a significant genetic differentiation between XCSS and the other five main stream local populations using mtDNA. This result suggested that gene flow between the XCSS and downstream local populations has been inhibited for a long time. This conclusion is supported by the YFDE2006 and YFDE2012, which observed a distribution gap area within an approximately 150 km stretch between Shishou and Yueyang (Figure 1). Furthermore, the genetic diversity analysis revealed that the restricted gene flow combined with the population decline in the XCSS may have caused a serious loss of genetic diversity, as the haplotype diversity (*h*) and nucleotide diversity (π) of the XCSS were zero. Compared to other local populations, the microsatellite diversity of the XCSS was also relatively low based on number of alleles detected per locus (*N*) and the allelic richness (*AR*) (Table 2). On the other hand, the microsatellite analysis revealed that significant genetic differentiation also appeared between the TL and local populations upstream according to the *F*st values (Table 3). No significant genetic differentiation was detected between the HHPZ and EZHS populations, suggesting adequate gene flow over the last several generations. However, restricted gene flow and consequent genetic differentiation will likely form between the HHPZ and EZHS populations, as a new gap of approximately 150 km surrounding Wuhan was reported by the YFDE2012 (Figure 1).

The Poyang and Dongting Lakes are two important limnic habitats of the YFP; in particular, Poyang Lake has a population size almost equal to the total population size in the Yangtze main stream. In the past ten years, overly dense human activities in the estuary area of these two lakes have largely inhibited the lake-river migrations of the YFP [9,10]. Thus, the genetic differentiation between the two lakes and the Yangtze main stream is particularly noteworthy. Our results indicated that the PY population was significantly differentiated from all other local populations, as revealed by the *F*st values from the microsatellite DNA analysis (Table 3). Because the *F*st reflects long-term gene flow [27], in theory, the development of significant genetic differentiation would be impossible within the ten years of human impact on the YFP, which has a generation time of eight years [28]. Therefore, we inferred that the large-scale lake-river migration of the YFP reported by Wei *et al*. [11] likely only occurred between the Hukou area and adjacent Yangtze main stream, whereas gene flow between the populations in the main stream and the main lake region, which connects to the main stream by a 67 km-long channel, has likely been low for a longer period of time. Although human activities may not be the cause of the genetic differentiation detected in this study, recent human-induced restriction on the lake-river migration in Hukou will eventually cause further differentiation of the PY population from the main stream population. The DT population faced a similar situation as the PY population. Our results indicated that although the DT was significantly differentiated from the XCSS, PY, and TL populations, no significant genetic differentiation was observed among the DT, HHPZ, and EZHS populations, suggesting that a certain level of gene flow has remained between the DT, HHPZ, and EZHS populations over the last several generations. However, in the past years, an increased density of human activities around the estuary area of the Dongting Lake has largely inhibited porpoise dispersal between the Dongting Lake and the Yangtze main stream [10], which may cause new genetic differentiation between the DT and main stream populations in the future. Overall, the YFP is in danger of becoming a genetically fragmented population.

## 4. Experimental Section

### 4.1. Sample Collection and DNA Extraction

A total of 186 porpoises were sampled between 1998 and 2011 (146 blood samples and 40 tissue samples) across the distribution range, including three from Shanghai (SH), 17 from Tongling (TL), five from Anqing (AQ), 91 from the Poyang Lake (PY), 15 from Ezhou–Huangshi (EZHS), three from Wuhan (WH), 14 from Honghu–Paizhou (HHPZ), 20 from the Dongting Lake (DT), and 18 from Xingchang–Shishou (XCSS) (Figure 1). Blood samples were drawn from the caudal vein of live captured porpoises when population structure surveys had been conducted in the Poyang Lake, or when live stranding porpoises had been rescued in the wild. Blood sample was immediately anti-coagulated with Acid-citrate-dextrose (ACD) solution and preserved in liquid nitrogen. All tissue samples (muscle or liver) were collected from dead stranding animals, and were preserved in 70% alcohol. All sampling was conducted in accordance with the Regulations of the People’s Republic of China for the Implementation of Wild Aquatic Animal Protection (promulgated in 1993), adhering to all ethical guidelines and legal requirements in China. Six local populations (TL, PY, EZHS, HHPZ, DT, and XCSS) were used in the subsequent population genetic analyses, while SH, AQ and WH were excluded because of their small sample size of less than five individuals.

Total genomic DNA was isolated from blood samples using the Whole Genome DNA Extraction Kit (SBS, Shanghai Inc., Shanghai, China) according to the manufacturer’s instructions. For tissue samples, total genomic DNA was extracted using standard proteinase K digestion and phenol/chloroform extraction protocols [29].

### 4.2. MtDNA Sequencing and Microsatellite Genotyping

A mtDNA control region segment of 597 base pair was amplified with the forward (5'-GAA TTC CCC GGT CTT GTA AAC C-3') and the reverse primers (5'-GGT TTG GGC CTC TTT GAG AT-3') designed in the present study by Primer Premier 5.0 (Premier Biosoft, Palo Alto, CA, USA). The PCR amplification was carried out in a 25 μL reaction containing 10–100 ng of genomic DNA, 2.5 μL of 10× buffer, 0.6 μM of each primer, 0.25 mM dNTPs and 1 U of Taq DNA polymerase (Biostar, Saskatoon, SK, Canada). The amplifications were carried out as follows: 94 °C for 5 min, followed by 35 cycles of denaturation at 94 °C for 45 s, annealing at 60 °C for 45 s, and extension at 72 °C for 90 s, and a final extension step at 72 °C for 7 min. The PCR products were purified and then sequenced on an ABI3130 DNA sequencer (Applied Biosystems, Foster City, CA, USA).

Eleven polymorphic and steadily amplified loci were used in this study: YFSSR1, YFSSR5, YFSSR40, YFSSR42, and YFSSR59 from *N*. *p*. *asiaeorientalis* [30,31]; NP391, NP404, NP409, NP428, and NP464 from *N*. *phocaenoides* [32,33]; and PPHO130 from *Phocoena phocoena* [20]. Each forward primer was labeled with the fluorescent dye 6-FAM at the 5' end. The PCR was performed in a 15 μL reactions containing 10–100 ng of genomic DNA, 1.5 μL of 10× buffer, 0.7 μM of each primers, 0.25 mM dNTPs and 0.2 U of Taq DNA polymerase (Biostar, Canada). The amplifications were carried out as follows: 95 °C for 5 min, followed by 33 cycles of denaturation at 95 °C for 30 s, annealing at 59.5 °C for 30 s, and extension at 72 °C for 30 s, and a final extension step at 72 °C for 5 min. The PCR products were separated by capillary electrophoresis using a denaturing acrylamide gel matrix on an ABI3130XL automated sequencer (Applied Biosystems). The alleles were sized against the internal size standard (GeneScan ROX500) using GeneMapperID version 3.2 (Applied Biosystems), and then were checked by eyes according to the genotyping map. To minimize scoring error, those samples that have homozygote, low frequency alleles (only appeared in one or two individuals) or stutter bands were amplified and genotyped at least three times. MICRO-CHECKER version 2.2.3 [34] was used to check for null alleles, stuttering error, and allele dropout at each locus.

### 4.3. Genetic Diversity Analysis

For mitochondrial DNA, all mtDNA control region sequences were analyzed with ClustalX [35,36]. The haplotype diversity (*h*) and nucleotide diversity (π) [37] of the entire population were calculated using DnaSP version 5 [38]. For microsatellite DNA, GENEPOP version 4.0 [39] was used to estimate the microsatellite diversity, including the observed and expected heterozygosity (*H*o and *H*e) and the number of alleles. FSTAT version 2.9.3.2 [40] was used to calculate the allelic richness (*AR*) and inbreeding coefficient (*F*is). Hardy-Weinberg equilibrium was assessed across all microsatellite loci for each local population via an exact probability test implemented in GENEPOP version 4.0. The significance values were adjusted for multiple comparisons using the Bonferroni correction. To detect whether the population suffered a rapid reduction in allele numbers, BOTTLENECK version 1.2.02 [41] was used to test for an excess of heterozygotes. Heterozygote excess was tested using a stepwise mutation model (SMM) and a two-phase mutation model (TPM), which are more appropriate models for microsatellite evolution [42]. Under the TPM model, the parameters were set to 95% single-step mutations and 5% multiple-step mutations, with a variance among multiple steps set to 12 (suggested by Piry *et al*. [43]). A one-tailed Wilcoxon sign test was used to test for statistical significance based on 10,000 iterations. A mode-shift test was also carried out using BOTTLENECK version 1.2.02 to detect a distortion of the expected L-shaped distribution of allele frequency (*i.e.*, lack of low-frequency alleles) for each local population [44].

### 4.4. Genetic Differentiation and Population Structure Analysis

To estimate the degree of genetic differentiation between the local populations, the *F*st was calculated using ARLEQUIN version 3.0 based on both the mtDNA and microsatellite DNA, and tested for significance with 10,000 permutations [45,46]. Next, a Bayesian model-based clustering method STRUCTURE version 2.1 was used to detect a possible subpopulation division across the sampling localities using microsatellite data [47]. STRUCTURE divides individuals into K clusters within which Hardy-Weinberg equilibrium and linkage equilibrium are achieved. The program uses a Markov chain Monte Carlo (MCMC) method to estimate Ln *P*(*D*), the probability of the data. The most likely number of subpopulations was taken when the Ln *P*(*D*) reached a plateaus. We chose an admixture model because it is more feasible to consider complex problems of real populations and hybrid zones in a natural manner [47]. STRUCTURE was run without original sampling information for all 186 individuals. A 100,000 MCMC burn-in period was carried out followed by 1,000,000 MCMC replicates per *K* from 1–10. Five independent runs were performed for each *K*. An ad hoc statistic Δ*K* was also used to help to find the best *K* [18].

Mantel test [48] was also performed to test significance of genetic isolation by geographical distance [49]. The geographical distance for populations was compared to *F*st/(1 − *F*st) in order to get a linear relationship. Mantel test was performed using ARLEQUIN version 3.0 and 10,000 iterations were used to determine the statistical significance of the results [46].

### 4.5. Simulations on the Evolution of Genetic Diversity

The conservation goal of maintaining 90% of the initial genetic diversity over a 100-year period [12] is arguably a more realistic objective than the optimal *N*e of at least 500, which was recommended by Franklin and Frankham [50] to maintain genetic diversity and avoid inbreeding depression. BOTTLESIM is a program specifically designed to simulate the evolution of the genetic diversity in long-lived species with overlapping-generations and to estimate the sustainable population size needed to meet the long-term conservation objective [51]. Estimations were performed with different *N*e values made temporally stable over a period of 150 years, with other simulation parameters held constant (lifespan = 20 years, age at maturity = 5 years [52,53], completely overlapping generations, dioecious reproduction, random mating, sex ratio of 1:1, 150 years simulated, 1000 iterations).

## 5. Conclusions and Conservation Implications

In summary, by using combined mtDNA and microsatellite markers, we have obtained a detailed understanding of the genetic diversity and population structure of the YFP. Although the two types of molecular marker revealed different levels of genetic diversities, they both suggested that the genetic diversity of the YFP will possibly decrease in the future if the population keeps declining rapidly. Regarding the population structure, the mitochondrial DNA indicated that significant genetic differentiation has appeared between the XCSS and the other five downstream local populations, whereas the microsatellite data unveiled more fine, but significant, genetic differentiation, which divided the entire population into four groups: XCSS, DT–HHPZ–EZHS, PY, and TL (Figure 3). Furthermore, we inferred that the genetic differentiation of the YFP will likely be aggravated in the future along with the appearance of additional distribution gaps, as discovered by recent ecological surveys.

Genetic diversity provides the raw material for the adaptation to environmental change [12,54]. Loss of genetic variation and an increased probability of inbreeding depression may interact with the demographic process to cause extinction of small fragmented populations [55,56]. Our study suggested that the YFP population will suffer from not only genetic diversity loss but also genetic differentiation between some local populations, which may cause further genetic diversity loss and increase the risk of extinction of the small local populations. Therefore, we recommend that further conservation efforts should also focus on the genetic conservation of this species. First, it is urgent to employ an effective strategy to prevent the further loss of its genetic diversity. The implications of this study suggest that more effort should be focused on protecting the porpoises in the middle section (EZHS, HHPZ, and DT), where the genetic diversity was higher than other local populations based on both mtDNA and microsatellite loci (allelic richness, *AR*). In addition, special conservation consideration should be given to the porpoise populations living in the Poyang and Dongting Lakes. Our analysis indicated that the porpoises in the two lakes contained 50% of the mtDNA diversity and 85% of the microsatellite allelic diversity. Carrying out effective protection of these two local populations will yield significant effects on the genetic diversity conservation of the entire population.

Second, effective conservation measures, such as habitat restoration and translocation, should be carried out to facilitate more widespread gene flow. This approach could not only reduce the loss of genetic diversity but also reduce the extinction risk of the small local populations because significant genetic differentiations have been detected between some local populations (XCSS, DT–HHPZ–EZHS, PY, and TL), and additional genetic differentiation may occur in the Yangtze main stream, where distribution gaps have appeared. Furthermore, the Poyang and Dongting Lakes are undoubtedly a crucial habitat that connects the different local populations in the Yangtze main stream, thus restricting human activities in the mouth area of these two lakes to restore the lake-river migration of the YFP will greatly benefit gene flow across the entire distribution range.

Human activities have caused extinction to the Yangtze River dolphin (*Lipotes vexillifer*) [57,58]. A similar fate will undoubtedly face the YFP, unless conservation management actions are taken. While the genetic data offer some important insights for the genetic conservation and management of the YFP, many other anthropogenic factors including over-fishing of prey species, water development projects that cause habitat loss and degradation, water pollution, and accidental deaths caused by harmful fishing gear and collisions with motorized vessels must be considered when developing a comprehensive conservation plan for the critically endangered YFP.

## Supplementary Files

Supplementary File 1
