# Supplementary material for: Genetic Diversity and Population Structure of the Critically Endangered Yangtze Finless Porpoise (Neophocaena asiaeorientalis asiaeorientalis) as Revealed by Mitochondrial and Microsatellite DNA
Source: Int J Mol Sci. 2014 Jun 25;15(7):11307–23. doi: 10.3390/ijms150711307 (PMC4139784; doi:10.3390/ijms150711307)
Supplement: Supplementary File 1 [file ijms-15-11307-s001.pdf]

Supplementary Information

Table S1. Likelihood of the number of population clusters, K. Columns 3 and 4 summarize the likelihoods and associated variances for different runs.

	**Independent Runs**	**Ln *P*(*D*)**	**Var [Ln *P*(*D*)]**
	Run1	−5652.3	41.8
	Run2	−5652.5	42.2
***K* = 1**	Run3	−5652.2	41.5
	Run4	−5652.2	41.4
	Run5	−5652.7	42.3
	Run1	−5656.3	376.8
	Run2	−5711.2	449.5
***K* = 2**	Run3	−5692.8	441.5
	Run4	−5683.2	421.4
	Run5	−5657.6	379.4
	Run1	−5614.5	462.6
	Run2	−5622.9	475.7
***K* = 3**	Run3	−5624.0	483.0
	Run4	−5608.4	442.8
	Run5	−5612.6	459.6

Figure S1. Results of STRUCTURE analyses. (**a**) Value of Ln *P*(*D*) from 10 independent runs for *K* = 1–10; (**b**) Value of *K* as a function of Δ*K* based on 10 runs.

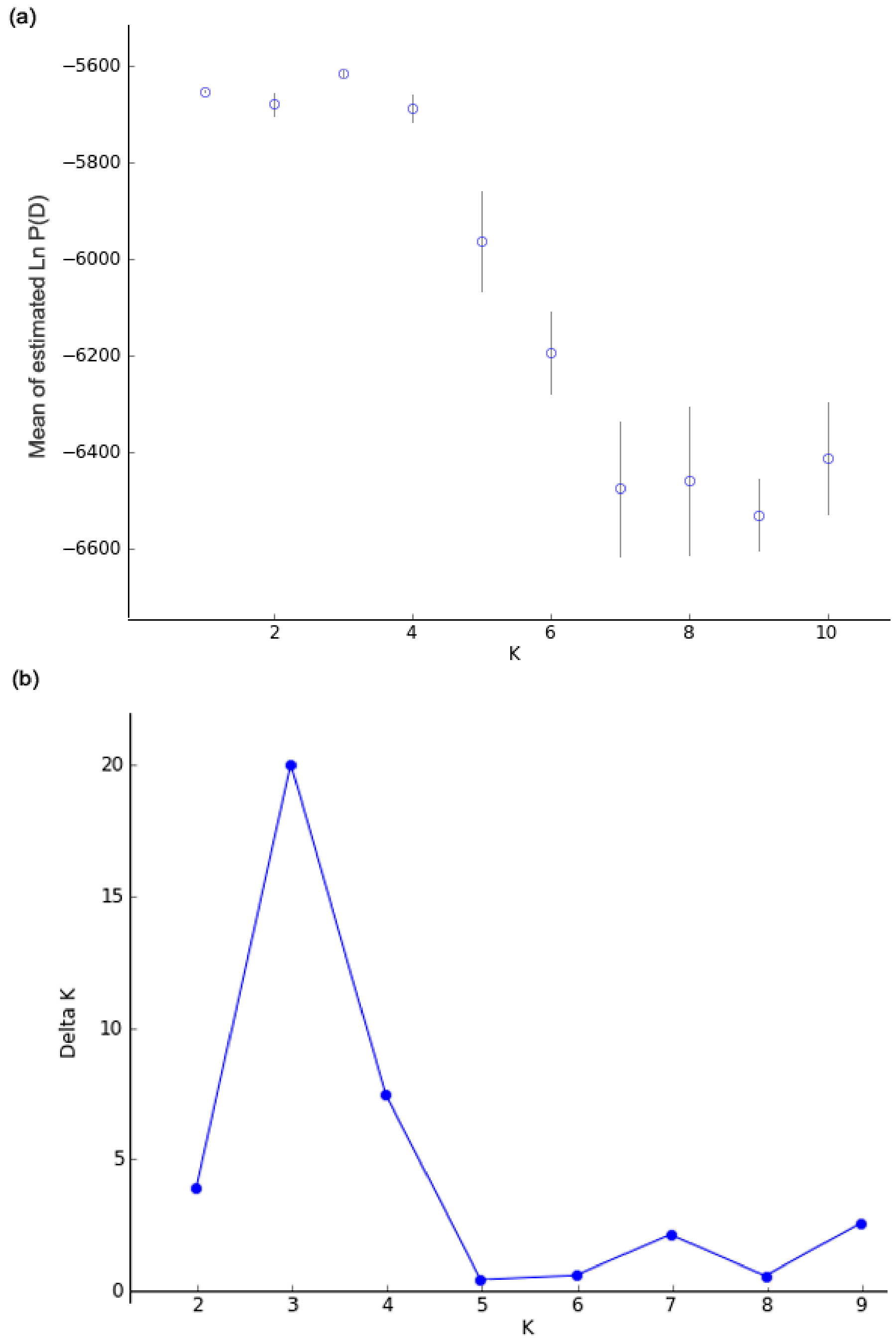

Figure S2. Estimated population structure when *K* = 2 and *K* = 3. Each individual is represented by a vertical line. The different local populations are separated by black lines. The population units are labeled in numbers (1: PY, 2: SH, 3: TL, 4: AQ, 5: EZHS, 6: WH, 7: HHPZ, 8: DT, 9: XCSS).

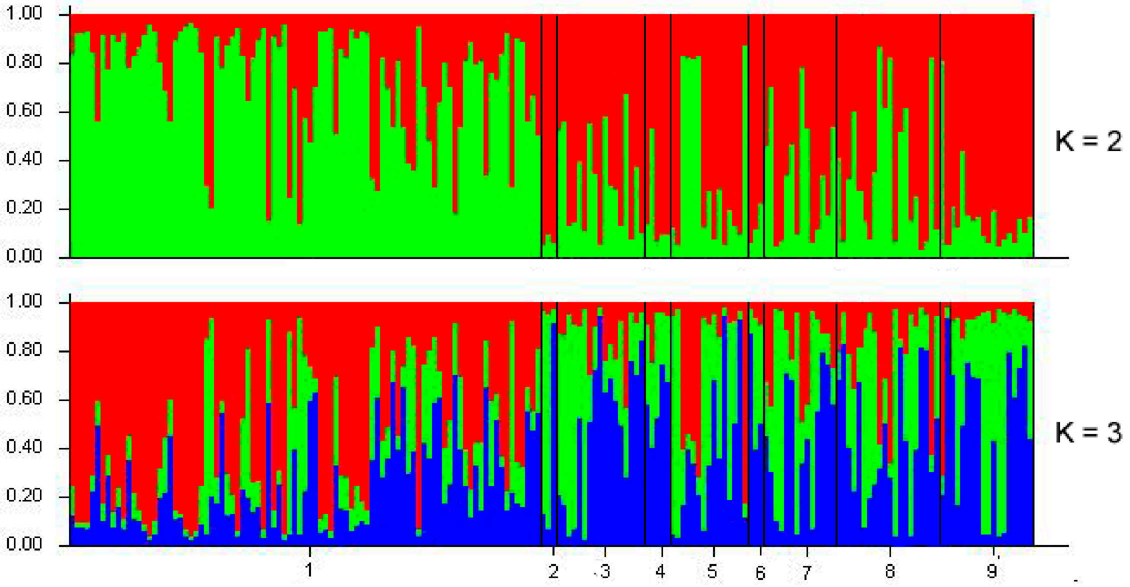

Table S2. Genetic diversity of each locus in each local population.

	TL					PY					EZHS				
**LOCUS**	*N*	*AR*	*H*o	*H*e	*F*is	*N*	*AR*	*H*o	*H*e	*F*is	*N*	*AR*	*H*o	*H*e	*F*is
**YFSSR1**	6	5.47	0.706	0.635	−0.032	5	4.039	0.633	0.621	−0.003	3	2.953	0.385	0.394	−0.062
**YFSSR5**	6	5.62	0.857	0.771	−0.114	9	6.024	0.759	0.816	0.071	6	5.981	0.813	0.822	−0.000
**YFSSR40**	7	5.643	0.824	0.722	−0.145	9	4.724	0.750	0.734	−0.073	5	4.962	0.736	0.695	−0.073
**YFSSR42**	3	2.588	0.412	0.415	0.009	5	4.035	0.611	0.67	0.124	4	3.963	0.642	0.538	−0.120
**YFSSR59**	6	5.525	0.706	0.818	0.141	8	6.351	0.912	0.837	−0.07	7	6.846	0.786	0.815	0.037
**PPHO130**	7	5.955	0.824	0.779	−0.059	8	5.626	0.786	0.802	−0.009	8	7.746	0.764	0.802	0.066
**NP391**	3	2.787	0.294	0.269	−0.096	8	3.104	0.220	0.244	0.080	7	6.793	0.515	0.569	−0.085
**NP404**	3	2.970	0.733	0.600	−0.232	4	3.033	0.527	0.550	0.066	4	3.788	0.462	0.433	−0.044
**NP409**	8	6.095	1.00	0.827	−0.217	8	5.529	0.667	0.757	0.104	4	3.967	0.615	0.701	0.127
**NP428**	6	4.497	0.467	0.620	0.254	5	3.504	0.378	0.367	−0.001	5	4.881	0.806	0.622	−0.382
**NP464**	4	3.639	0.867	0.607	−0.444	6	4.206	0.640	0.683	0.063	5	4.688	0.615	0.556	−0.091

	HHPZ					DT					XCSS				
**LOCUS**	*N*	*AR*	*H*o	*H*e	*F*is	*N*	*AR*	*H*o	*H*e	*F*is	*N*	*AR*	*H*o	*H*e	*F*is
**YFSSR1**	5	4.722	0.769	0.692	−0.116	5	4.768	0.657	0.765	−0.046	5	4.333	0.8	0.717	−0.120
**YFSSR5**	6	5.977	0.843	0.805	−0.259	6	5.780	0.865	0.756	−0.143	5	4.727	0.545	0.649	0.166
**YFSSR40**	6	5.347	0.709	0.695	−0.111	6	5.327	0.75	0.718	−0.134	6	5.533	0.867	0.788	−0.103
**YFSSR42**	4	3.908	0.529	0.643	−0.206	4	3.862	0.796	0.679	−0.138	4	3.536	0.438	0.383	−0.148
**YFSSR59**	7	6.933	0.733	0.869	0.043	8	7.585	0.893	0.879	−0.138	7	6.266	0.867	0.786	−0.106
**PPHO130**	9	8.963	0.841	0.905	−0.005	10	7.572	0.849	0.705	−0.122	8	6.317	0.786	0.745	−0.088
**NP391**	5	4.753	0.452	0.407	−0.124	6	4.385	0.333	0.471	0.256	5	4.534	0.667	0.651	−0.026
**NP404**	4	3.000	0.682	0.647	−0.086	3	2.683	0.6	0.568	−0.158	4	3.889	0.667	0.623	−0.073
**NP409**	5	4.909	0.616	0.784	0.195	6	5.663	0.627	0.818	0.101	5	3.896	0.737	0.725	0.069
**NP428**	4	3.996	0.515	0.619	0.124	5	4.447	0.732	0.585	−0.309	3	2.724	0.426	0.362	−0.191
**NP464**	5	4.818	0.602	0.658	0.034	8	5.951	0.717	0.775	0.040	6	4.647	0.857	0.712	−0.214
